# Microarray analysis of subcutaneous adipose tissue from mature cows with divergent body weight gain after feed restriction and realimentation

**DOI:** 10.1016/j.dib.2017.10.016

**Published:** 2017-10-10

**Authors:** H.C. Cunningham, K.M. Cammack, K.E. Hales, H.C. Freetly, A.K. Lindholm-Perry

**Affiliations:** a University of Wyoming, Laramie, WY 82071, USA; b USDA, ARS, US Meat Animal Research Center, P.O. Box 166, Clay Center, NE 68933, USA; c South Dakota State University, West River Ag Center, Rapid City, SD 57702, USA

**Keywords:** Beef cows, Subcutaneous fat, Transcriptome

## Abstract

Body weight response to periods of feed restriction and realimentation is critical and relevant to the agricultural industry. The purpose of this study was to evaluate differentially expressed genes identified in subcutaneous adipose tissue collected from cows divergent in body weight (BW) gain after feed restriction and realimentation. We compared adipose samples from cows with greater gain based on average daily gain (ADG) during realimentation with samples from cows with lesser gain. Specifically, there were four comparisons including two comparing the high and low gain animals across each feeding period (feed restriction and realimentation) and two that compared differences in feed restriction and realimentation across high or low gain classifications. Using microarray analysis, we provide a set of differentially expressed genes identified between the high and low gain at both periods of nutrient restriction and realimentation. These data identify multiple differentially expressed genes between these two phenotypes across both nutritional environments.

**Specifications Table**

**Table:** 

Subject area	*Biology*
More specific subject area	*Livestock transcriptomics*
Type of data	*Table and figures*
How data was acquired	*Affymetrix Bovine 1.1*^*ST*^*Gene Array (Microarray technology)*
Data format	*Filtered, analyzed*
Experimental factors	*RNA isolated from adipose tissue collected from the same cows following exposure to two nutritional treatments; feed restriction and realimentation.*
Experimental features	*Transcriptomic analysis of subcutaneous adipose tissue from cows divergent in body weight gain following two nutritional treatments.*
Data source location	*USDA-ARS, U.S. Meat Animal Research Center, Clay Center, NE, USA*
Data accessibility	*Data is accessible through the NCBI GEO database. The series record ID is GSE94746 located at*https://www.ncbi.nlm.nih.gov/geo/query/acc.cgi?acc=GSE94746

**Value of the data**

• Body weight gain is a complex trait closely connected with feed efficiency; thus, identifying gene networks and metabolic pathways potentially involved in the divergence of weight gain may provide a platform for further investigation into some of the critical control points of feed efficiency.
• Adipose tissue is a highly metabolically active tissue and also is closely regulated by energetics of the animal. Gene networks identified in this tissue may provide insight into how, during extreme energy balance times, animals divergent in body weight gain respond and adjust to these nutritional extremes.
• Differentially expressed genes and pathways identified in these comparisons may be used in future experiments investigating response in adipose tissue to nutritional status and divergence in feed efficiency.
• Datasets evaluating the molecular mechanisms of feed restriction and realimentation in cattle are scarce; thus, these data may be useful for inclusion with additional sets of similar data for a meta-analysis of nutritional treatments.

## 1 Data

**Fig. 1:**
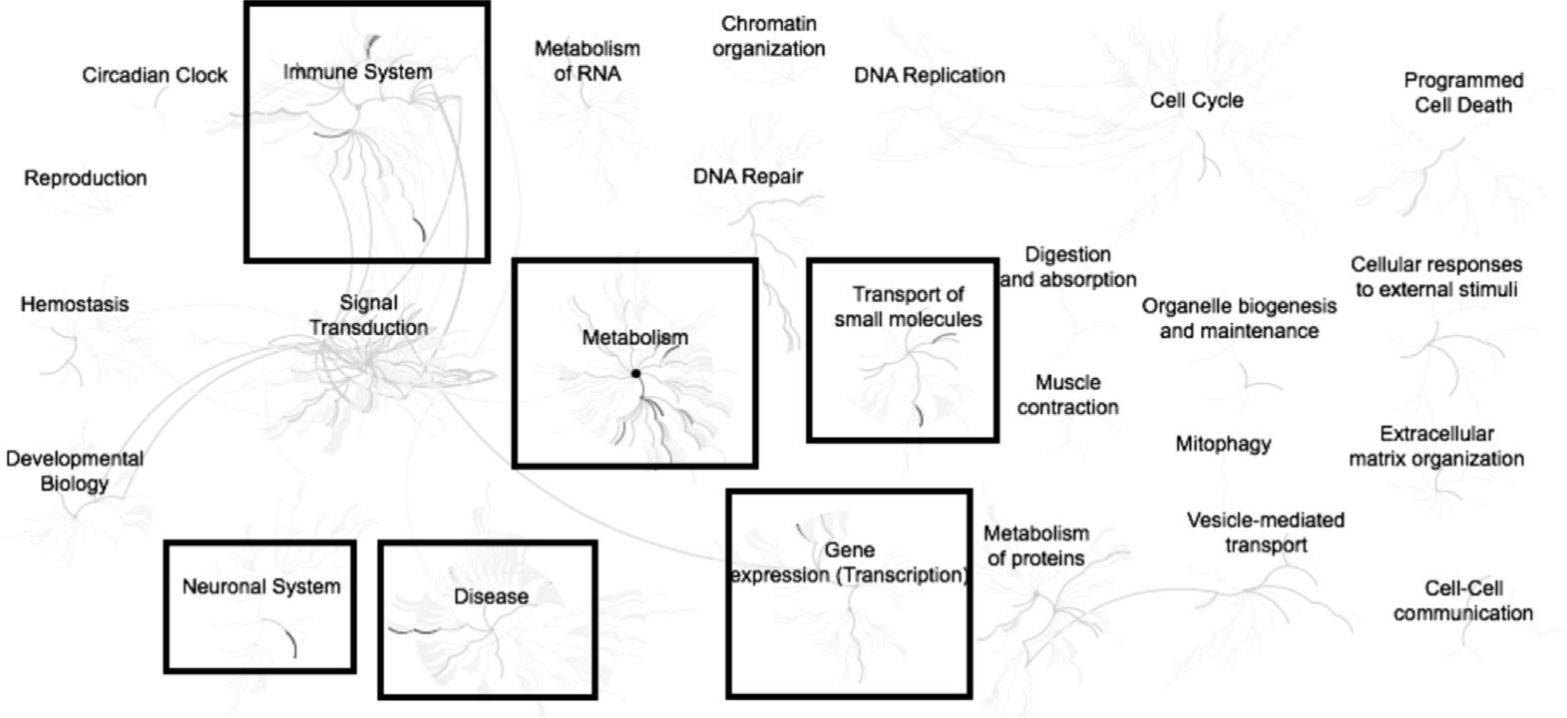
Enriched pathways from the list of genes (n = 68) down-regulated in adipose samples during feed restriction in high gaining animals compared to the low gaining animals. Overrepresented pathways (n = 23) are organized into overarching nodes, and the size of the node correlates with the numbers of entities belonging to the pathway. Edges represent the connection between pathways. The central node represents the top-level pathway with sub-level nodes project outwards from the central node. All pathways significantly (P < 0.05) overrepresented are indicated in bold black. Black boxes indicate which top-level and associated sub-pathways contain the significantly enriched pathways. The reactome pathway identifiers, pathway names, and genes identified in these enriched pathways are provided in the Supplementary material.

**Fig. 2:**
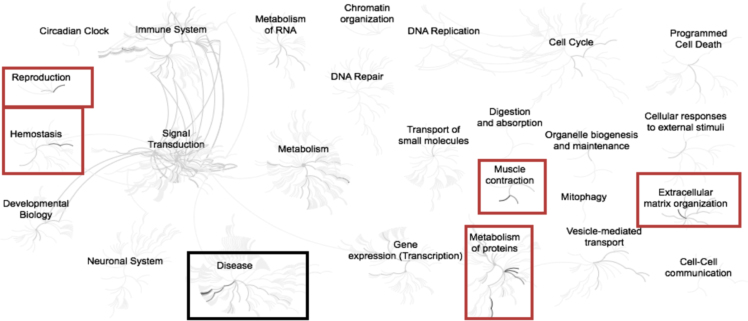
Enriched pathways from the list of list of genes (n = 45) up-regulated in adipose samples during feed restriction in high gaining animals compared to the low gaining animals. Overrepresented pathways (n = 21) are organized into overarching nodes, and the size of the node correlates with the numbers of entities belonging to the pathway. Edges represent the connection between pathways. The central node represents the top-level pathway with sub-level nodes project outwards from the central node. All pathways significantly (P < 0.05) overrepresented are indicated in bold black. Both black and orange boxes indicate which top-level and associated sub-pathways contain the significantly enriched pathways where the red boxes specifically indicate those enriched pathways identified distinct from the down-regulated genes. The reactome pathway identifiers, pathway names, and genes identified in these enriched pathways are provided in the Supplementary material.

**Fig. 3:**
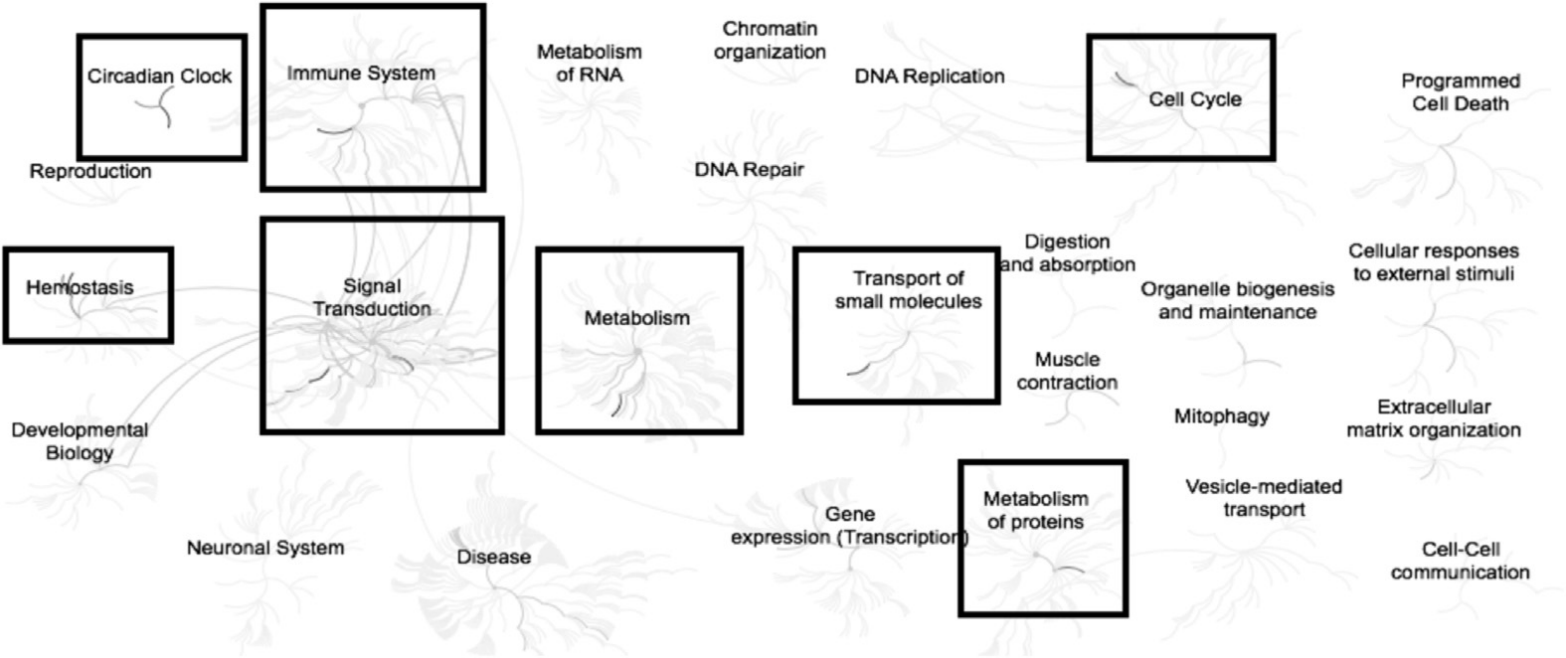
Enriched pathways from the list of genes (n = 27) down-regulated in adipose samples during realimentation in high gaining animals compared to the low gaining animals. Overrepresented pathways (n = 15) are organized into overarching nodes, and the size of the node correlates with the numbers of entities belonging to the pathway. Edges represent the connection between pathways. The central node represents the top-level pathway with sub-level nodes project outwards from the central node. All pathways significantly (P < 0.05) overrepresented are indicated in bold black. Black boxes indicate which top-level and associated sub-pathways contain the significantly enriched pathways. The reactome pathway identifiers, pathway names, and genes identified in these enriched pathways are provided in the Supplementary material.

**Fig. 4:**
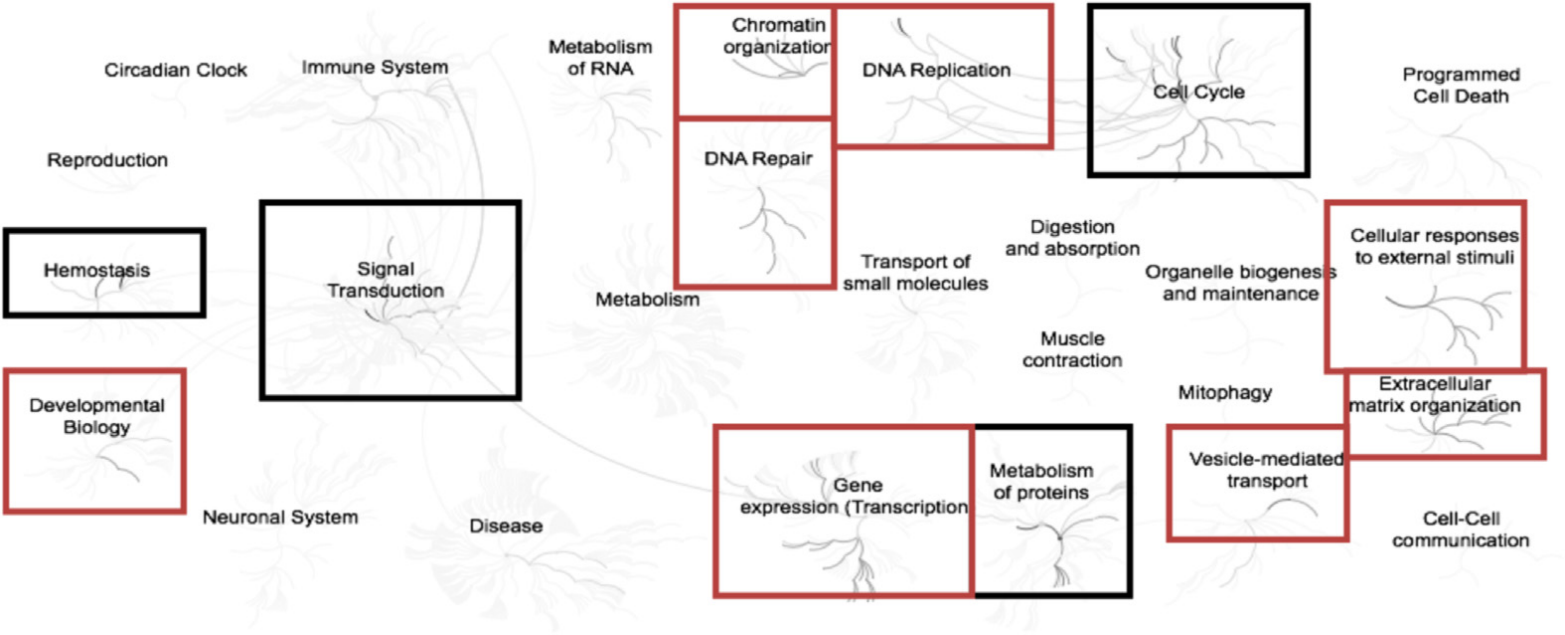
Enriched pathways from the list of list of genes (n = 33) up-regulated in adipose samples during realimentation in high gaining animals compared to the low gaining animals. Overrepresented pathways (n = 102) are organized into overarching nodes, and the size of the node correlates with the numbers of entities belonging to the pathway. Edges represent the connection between pathways. The central node represents the top-level pathway with sub-level nodes project outwards from the central node. All pathways significantly (P < 0.05) overrepresented are indicated in bold black. Both black and orange boxes indicate which top-level and associated sub-pathways contain the significantly enriched pathways where the red boxes specifically indicate those enriched pathways identified distinct from the down-regulated genes. The reactome pathway identifiers, pathway names, and genes identified in these enriched pathways are provided in the Supplementary material.

**Fig. 5:**
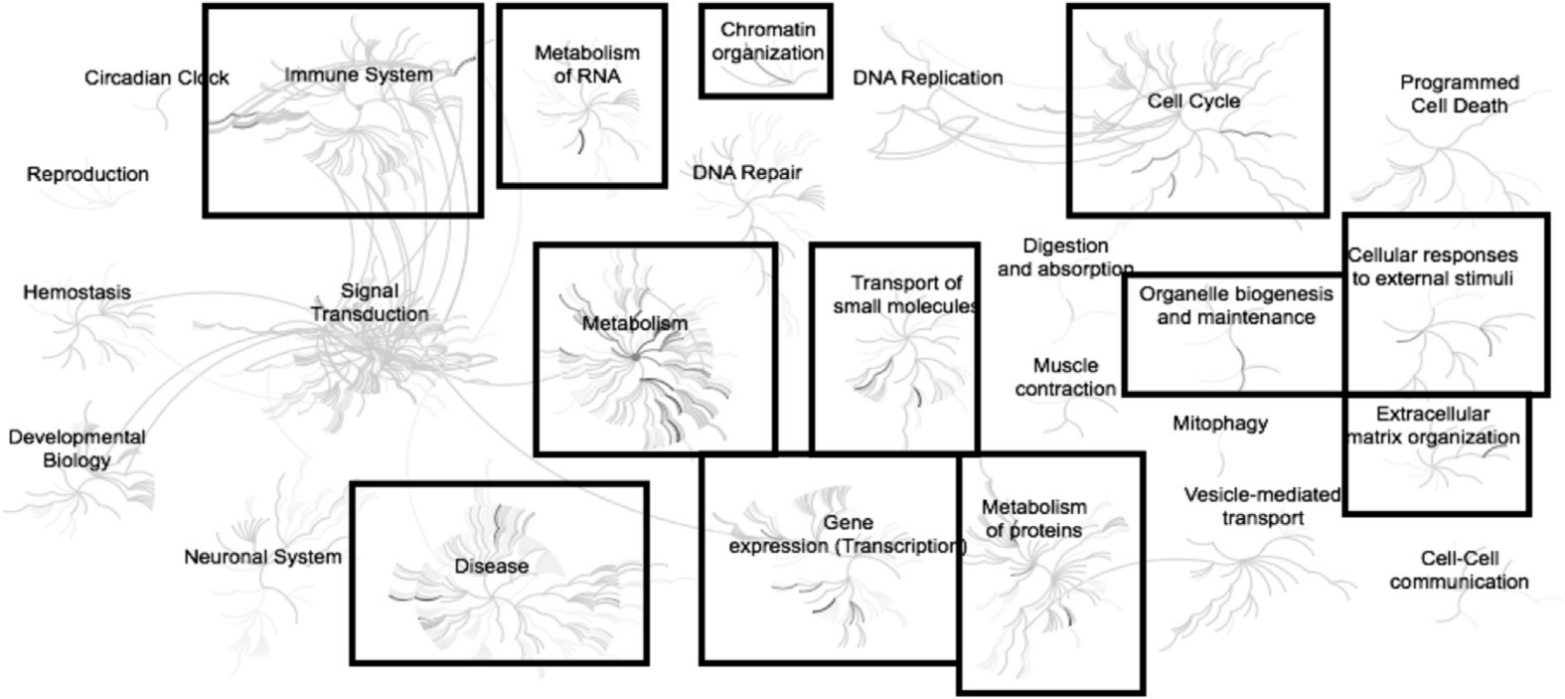
Enriched pathways from the list of genes (n = 433) down-regulated in adipose samples from low gaining animals during the feed restriction period compared to realimentation. Overrepresented pathways (n=79) are organized into overarching nodes, and the size of the node correlates with the numbers of entities belonging to the pathway. Edges represent the connection between pathways. The central node represents the top-level pathway with sub-level nodes project outwards from the central node. All pathways significantly (P<0.05) overrepresented are indicated in bold black. Black boxes indicate which top-level and associated sub-pathways contain the significantly enriched pathways. The reactome pathway identifiers, pathway names, and genes identified in these enriched pathways are provided in the Supplementary material.

**Fig. 6:**
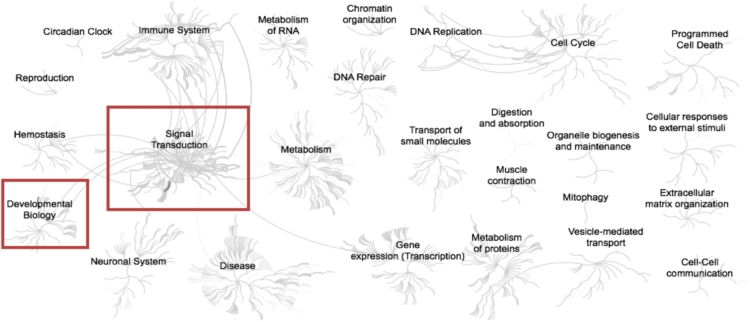
Enriched pathways from the list of list of genes (n=2041) up-regulated in adipose samples from low gaining animals during the feed restriction period compared to realimentation. Overrepresented pathways (n=2) are organized into overarching nodes, and the size of the node correlates with the numbers of entities belonging to the pathway. Edges represent the connection between pathways. The central node represents the top-level pathway with sub-level nodes project outwards from the central node. All pathways significantly (P<0.05) overrepresented are indicated in bold black. Both black and orange boxes indicate which top-level and associated sub-pathways contain the significantly enriched pathways where the red boxes specifically indicate those enriched pathways identified distinct from the down-regulated genes. The reactome pathway identifiers, pathway names, and genes identified in these enriched pathways are provided in the Supplementary material.

**Fig. 7:**
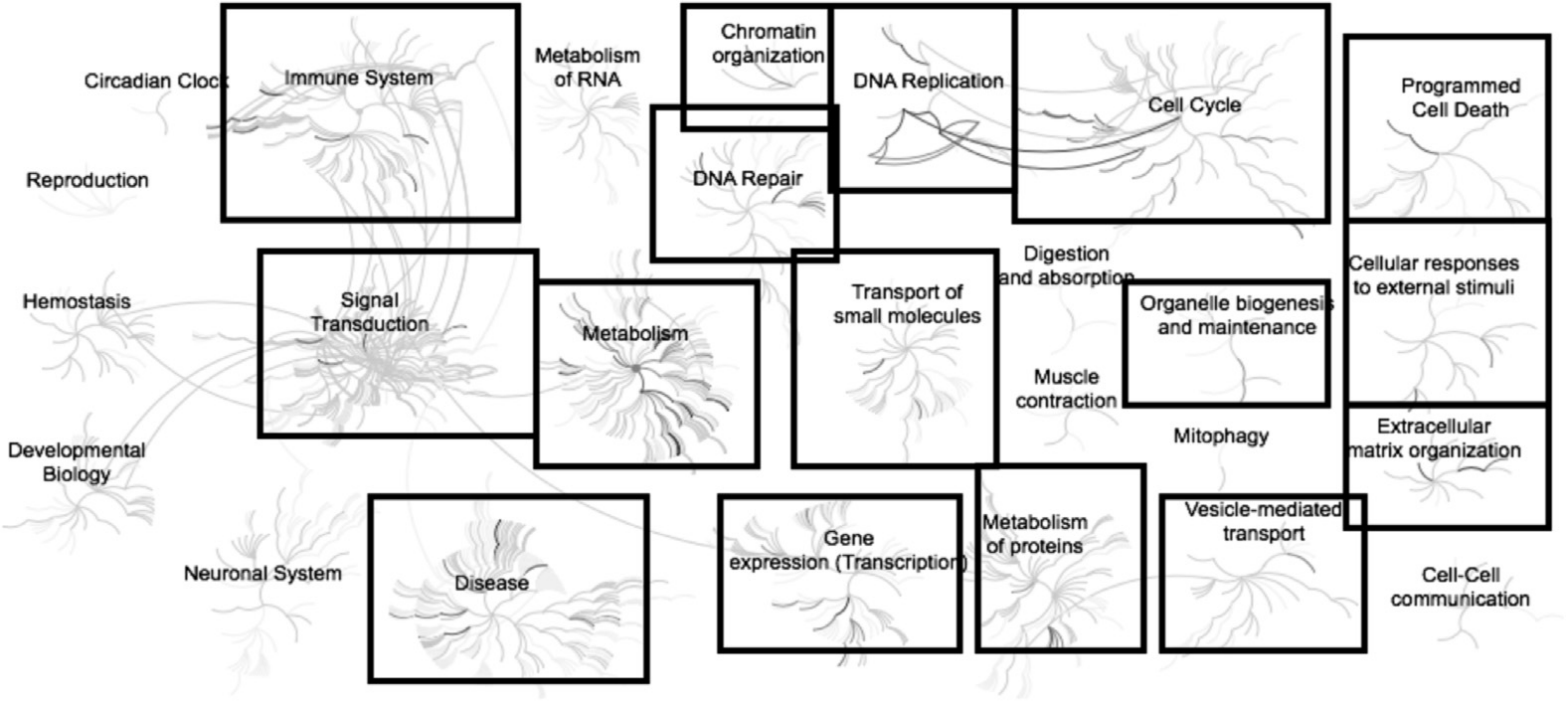
Enriched pathways from the list of genes (n=583) down-regulated in adipose samples from high gaining animals during the feed restriction period compared to realimentation. Overrepresented pathways (n=122) are organized into overarching nodes, and the size of the node correlates with the numbers of entities belonging to the pathway. Edges represent the connection between pathways. The central node represents the top-level pathway with sub-level nodes project outwards from the central node. All pathways significantly (P<0.05) overrepresented are indicated in bold black. Black boxes indicate which top-level and associated sub-pathways contain the significantly enriched pathways. The reactome pathway identifiers, pathway names, and genes identified in these enriched pathways are provided in the Supplementary material.

**Fig. 8:**
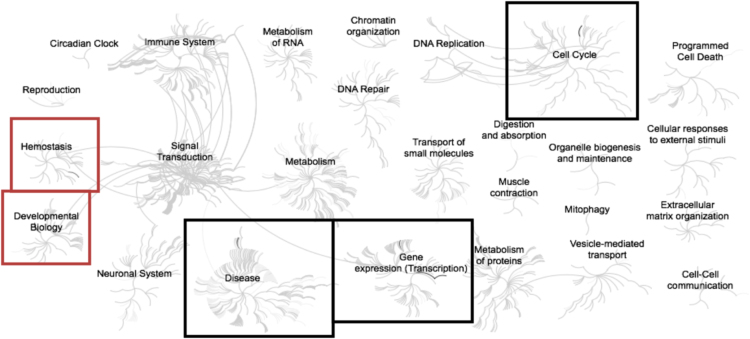
Enriched pathways from the list of list of genes (n=2295) up-regulated in adipose samples from high gaining animals during the feed restriction period compared to realimentation. Overrepresented pathways (n=7) are organized into overarching nodes, and the size of the node correlates with the numbers of entities belonging to the pathway. Edges represent the connection between pathways. The central node represents the top-level pathway with sub-level nodes project outwards from the central node. All pathways significantly (P<0.05) overrepresented are indicated in bold black. Both black and orange boxes indicate which top-level and associated sub-pathways contain the significantly enriched pathways where the red boxes specifically indicate those enriched pathways identified distinct from the down-regulated genes. The reactome pathway identifiers, pathway names, and genes identified in these enriched pathways are provided in the Supplementary material.

Microarray analysis comparing mRNA isolated from subcutaneous adipose of 12 cows (6 high ADG and 6 low ADG) collected at two nutritional stages (nutrient restricted vs. ad libitum). This results in four separate comparisons; 1) Feed-restricted treatment: high vs. low gain cows; 2) Ad libitum treatment: high vs. low gain cows; 3) Low gain cows: feed-restricted vs. ad libitum treatments; 4) High gain cows: feed-restricted vs. ad libitum treatments. A list of differentially expressed genes (> 2.0-fold; nominal *P* < 0.05) was generated for each comparison (Supplementary material). Pathway analysis for the overrepresented down-regulated and up-regulated gene lists identified enriched pathways and relationships between pathways for each comparison (Fig. 1, Fig. 2, Fig. 3, Fig. 4, Fig. 5, Fig. 6, Fig. 7, Fig. 8). For comparison 1, a total of 68 (61 annotated) down-regulated and 45 (39 annotated) up-regulated differentially expressed genes were used in pathway analyses with a total of 4 and 9 nodes identified containing 23 and 21 pathways, respectively (Fig. 1, Fig. 2). In comparison 2, 27 (24 annotated) and 33 (30 annotated) differentially expressed genes resulted in 6 (15 pathways) and 13 (102 pathways) nodes identified for the down-regulated and up-regulated lists, respectively (Fig. 3, Fig. 4). A total of 433 (412 annotated) down-regulated and 2041 (1556 annotated) up-regulated genes were differentially expressed in comparison 3 and resulted in 16 and 2 nodes containing 79 and 2 pathways, respectively (Fig. 5, Fig. 6). In the final comparison, 583 (551 annotated) down-regulated genes resulted in 8 overrepresented nodes containing 122 pathways and 2295 (1917 annotated) up-regulated genes resulted in 2 nodes containing 7 pathways (Fig. 7, Fig. 8). These data are consistent with gene ontology analysis [1].

## 2 Experimental design, materials and methods

### 2.1 Animal management and diets

Crossbred cows (n=121) that were the result of sampling sires used in the beef industry were used in the study. Angus, Hereford, and MARC III composite (¼ Angus, ¼ Hereford, ¼ Pinzgauer, ¼ Red Poll) cows were bred by artificial insemination to Angus, Hereford, Simmental, Limousin, Charolais, Gelbvieh and Red Angus bulls. The F_1_ bulls from these matings from Angus and Hereford dams were mated to F_1_ cows from these matings in multiple-sire pastures to produce 2-, 3- and 4-breed cross progeny. The resulting female progeny were kept and raised to have their first calf at 2 years old. At 5 years of age, cows were not bred, and were moved to an individual feed intake facility equipped with Calan Gates (American Calan, Northwood NH) the week after their calves were weaned. Cows were fed a ration that contained 27.0% ground alfalfa hay, 5.0% corn, 67.8% corn silage, and 0.2% salt all on a dry-matter basis. Twenty-one days after weaning, cows were weighed on two consecutive days. Body weight was averaged and feed offered was set to provide 120 kcal ME/kg metabolic body weight (BW^0.75^). Cows were fed the same amount of feed for 112 days. At 112 days, cows had ad libitum access to the same ration for an additional 98 days. Cows were fed once a day and feed refusal was measured weekly. During the feed restriction, cows were weighed on days 0, 1, 14, 28, 56, 84, 111, and 112. During the ad libitum period, cows were weighed on days 0, 14, 28, 42, 56, 70, 84, 97, and 98. Individual body weight was regressed on time during the ad libitum period using a quadratic equation, and body weight gain over the study was calculated from the regression equation.

### 2.2 Tissue collection

Adipose biopsies were taken 105 days after the start of the feed restriction and 49 days after the start of the ad libitum feeding period. The sample site was scrubbed with betadine and rinsed with water. Twenty milliliters of lidocaine was injected subcutaneously at the incision site, and a 5-cm incision was made through the skin with a scalpel above the fat pad between the hooks and pins. Adipose tissue sample was harvested with iris scissors. The sample was immediately frozen in liquid nitrogen and stored at −80 °C. The wound was closed with Braunamid 4 USP suture (B. Braun AESCULAP, Germany), and sutures were removed 14 days later. The sample taken during restriction was from the left side of the cow, and the sample take during ad libitum feeding was taken from the right side of the animal.

Six cows with the greatest and 6 cows with the least body weight gain during the ad libitum period were selected and adipose tissue from these 12 animals was processed for microarray analysis. This resulted in 24 samples for microarray analysis (12 cows; 2 sampling times). Breed diversity among the high gain animals was: 25% Hereford, 16.7% Angus, 16.7% MARC III, 12.5% Simmental, 12.5% Gelbvieh, and 16.7% Red Angus. Breed composition among the low gain animals was: 25% Hereford, 16.7% Angus, 16.7% MARCIII, 4.2% Simmental, 4.2% Limousin, 4.2% Charolais, 4.2% Gelbvieh, and 25% Red Angus.

### 2.3 RNA isolation and quality assessment

Total RNA was extracted from 50 to 100 mg of tissue by homogenization with TriPure reagent (Roche, Indianapolis, IN) following the manufacturer's protocol with the exception of an increased time of centrifugation from 15 to 20 min following the addition of chloroform. RNA pellets were resuspended in sterile water and RNA was stored at -80°C until further processing. Purified RNA was quantified using a NanoDrop 8000 spectrophotometer (Thermo Scientific, Wilmington, DE). Absorbance was measured by spectrophotometry at 260 nm. The quality of total RNA was determined by running samples on a RNA 6000 LabChip kit (Agilent Technologies, Santa Clara, CA) with the Agilent 2100 bioanalyzer. Additionally, RNA electrophoresis was utilized to view the quality based on intensity of the 28 S and 18 S RNA. Average spectrophotometry measurement 260/280 ratios were 1.94 and 1.96 and average RIN values were 6.6 and 7.2 for adipose samples collected from cows at feed restriction and realimentation, respectively.

### 2.4 Microarray

To assess the differential expression of genes in adipose tissue, the Affymetrix GeneAtlas System (Santa Clara, CA) in conjunction with Bovine 1.1ST array strips were used. These arrays evaluate 24,341 genes using 526,810 probes. Briefly, RNA purity and yield was confirmed by spectrophotometry. One hundred ng of total RNA and controls were converted to single stranded cDNA, then fragmented and TdT labeled according to the WT Expression Kit manufacturer's instructions (Ambion®, Life Technologies Corporation). Chips were washed and stained in the GeneAtlas Fluidics station. Chips were immediately imaged in a calibrated Affymetrix GeneAtlas scanner. The CEL formatted files were converted to CHP files with Affymetrix Expression Console software [2]. Guanine Cytosine Count Normalization (GCCN) and Signal Space Transformation (SST) algorithms were applied to the microarray data. The GCCN program normalizes the signal of the probes by GC content. The SST algorithm was used to stretch the signal intensity distribution in order to decompress the fold change ratios. Samples were then subjected to Robust Multichip Analysis (RMA) for processing, further normalization and analysis of the microarrays [3]. Transformed data was analyzed with the Affymetrix Expression Console and Transcription Analysis Console software. Genes were considered differentially expressed when a p-value of <0.05 and a fold change of >±2 was obtained (Supplementary material). Data was deposited in the NCBI gene expression omnibus (GEO) under Series record GSE94746.

### 2.5 Pathway analysis

The differentially expressed gene lists were then further analyzed into down-regulated and up-regulated genes lists and used for overrepresentation pathway analyses using Reactome (v58) (http://www.reactome.org/) [4], [5]. Pathways overrepresented (*P*<0.05) were organized by node, and the relationship between nodes were generated for each gene list across each comparison (Fig. 1, Fig. 2, Fig. 3, Fig. 4 and Supplementary material).
